# Reply to Janssen et al. Comment on “Sobczyk, M.K.; Gaunt, T.R. The Effect of Circulating Zinc, Selenium, Copper and Vitamin K_1_ on COVID-19 Outcomes: A Mendelian Randomization Study. *Nutrients* 2022, *14*, 233”

**DOI:** 10.3390/nu14051113

**Published:** 2022-03-07

**Authors:** Maria K. Sobczyk, Tom R. Gaunt

**Affiliations:** MRC Integrative Epidemiology Unit, Bristol Medical School, University of Bristol, Bristol BS8 2BN, UK; tom.gaunt@bristol.ac.uk

In their correspondence arising from our recent manuscript [1], Janssen et al. [2] largely repeat the limitations we presented in the Discussion of our Mendelian randomization (MR) analysis that aimed to establish causal relationships between vitamin K_1_ levels and COVID-19 outcomes: infection, hospitalization, and a very severe disease course. In our manuscript we acknowledged that the MR analysis suffers from relatively low power influenced both by the power of exposure in the outcome GWAS, and “winners’ curse” in the exposure GWAS. We concluded that we find limited statistical evidence for vitamin K_1_’s role in COVID-19 (but do not claim that this lack of evidence is evidence of no effect): the point estimates in the main method (IVW) point to slightly reduced very severe COVID and hospitalizations (versus the general population) being associated with higher genetically predicted circulating vitamin K_1_ levels.

However, previous MR investigations using the same circulating phylloquinone GWAS for genetic instrument selection and effect-size estimates did uncover strong evidence for a modest causal effect on type 2 diabetes [3] and large artery atherosclerotic stroke [4]. This is why our manuscript encourages future investigations using better-powered exposure and outcome GWAS.

Furthermore, in our manuscript we concluded that effect estimates obtained in our MR analysis relate to the preventative effect of the naturally occurring range of circulating phylloquinone concentration prior to infection; moreover, we confirm that our analysis was not intended to mirror the potential therapeutic effect of high-dose clinical interventions which may be needed for seriously ill, hospitalized individuals.

Contrary to the implicit suggestion in Janssen et al. [2], there is no gold-standard biomarker for determining extrahepatic vitamin K status, with blood phylloquinone (genetically instrumented in our study), PIVKA-II and dp-ucMGP (used by Janssen and colleagues in their research) widely used and attributed to a specific set of strengths and disadvantages; this is reviewed in-depth elsewhere [5,6]. We acknowledge that genetic instruments for dp-ucMGP are available [7] and that additional MR analyses involving them could complement our study.

In their closing paragraph, Janssen et al. [2] state that: “we are of the opinion that their (i.e., Sobczyk & Gaunt 2022) genetic data analysis is interesting but cannot be used to decide whether vitamin K supplementation has a role in COVID-19”; this agrees with the sentiment expressed in our manuscript, as shown by the multiple limitations we described and our concluding statement about the lack of evidence from our analysis.

The previous track record of vitamins (such as D) and micronutrients (such as zinc) with plausible mechanistic pathways and much more accumulated observational evidence than vitamin K in randomized controlled trials (RCT) has not been encouraging [8]. This is why we await with interest the publication of results from the phase 2 trial on vitamin K_2_ supplementation in COVID-19, which is led by Janssen’s co-authors and industry collaborators (KOVIT trial, ClinicalTrials.gov Identifier: NCT04770740, estimated completion date October 2021), and includes pulmonary damage and coagulopathy as secondary outcomes. As with other medical interventions, vitamin supplementation can lead to side effects, and therefore, multiple strands of evidence (in vitro, animal models, RCTs etc.) may be needed to establish whether vitamin K has a role in prophylaxis and/or the therapy of COVID-19 before any clinical recommendations can be made; we certainly made no such recommendations in our own manuscript.
